# Case report: manic episode with psychotic symptoms induced by hyponatremia

**DOI:** 10.1007/s40211-020-00335-z

**Published:** 2020-02-11

**Authors:** Laurin Mauracher, Maria Rettenbacher

**Affiliations:** Department of Psychiatry, Psychotherapy and Psychosomatics, University Hospital for Psychiatry I, Medical University of Innsbruck, Anichstraße 35, 6020 Innsbruck, Austria

**Keywords:** Mania, Hyponatremia, Psychosis, Sodium, Polydipsia, Manie, Hyponatriämie, Psychose, Natrium, Polydipsie

## Abstract

In the literature, several cases of an association between hyponatremia and psychotic symptoms have been reported. We present the case of a young Caucasian male presenting with rapid, incoherent speech, religious and megalomanic delusions, and emotional lability. The patient was described by his relatives as being healthy until a few days before admission. He had no significant medical or psychiatric history, except a short drug-induced psychotic episode a few years earlier. Somatic workup showed moderate hyponatremia, but no other abnormalities. Tests for narcotics, in particular, were also negative. Antipsychotic treatment with risperidone was initiated. After normalization of sodium levels using intravenous saline, the patient remitted within a few days and risperidone was discontinued on day 3. He was discharged by day 13 without further pharmacological treatment.

Dysfunction of voltage-gated ion channels, particularly sodium and calcium channels, has been implicated in the pathogenesis of bipolar disorder. We therefore assume a causal relationship between hyponatremia and manic-psychotic symptoms in our patient. Hyponatremia was most likely induced by excessive water intake during a period of fasting in the context of a wellness practice.

## Abstract (de)

In der Literatur sind mehrere Fallberichte von PatientInnen mit Hyponatriämie-assoziierten psychotischen Symptomen beschrieben worden. Hier stellen wir den Fall eines jungen, kaukasischen Mannes vor, der mit manisch-psychotischer Symptomatik (beschleunigter, inkohärenter Gedankenduktus, wahnhafte religiöse und Größenideen sowie Affektlabilität) vorstellig wurde. Laut Fremdanamnese durch die Angehörigen sei der Patient bis wenige Tage vor der stationären Aufnahme unauffällig und gesund gewesen. Die Anamnese war negativ für psychiatrische oder relevante somatische Erkrankungen, abgesehen von einer kurzen drogeninduzierten psychotischen Episode einige Jahre zuvor. Die somatische Abklärung wies bis auf eine deutliche Hyponatriämie keine Auffälligkeiten auf. Das Drogenscreening war ebenfalls negativ. Risperidon wurde als Antipsychotikum verabreicht. Nach Normalisierung der Natriumkonzentration durch Infundierung von Kochsalzlösung klangen die Symptome innerhalb weniger Tage ab. Risperidon wurde nach drei Tagen wieder abgesetzt, und der Patient konnte nach 13 Tagen remittiert und ohne weitere medikamentöse Behandlung entlassen werden. In der Literatur wird eine Dysfunktion von spannungsgesteuerten Ionenkanälen, namentlich von Natrium- und Kalzium-Kanälen, mit der Pathogenese der bipolaren Störung in Verbindung gebracht. Wir vermuten bei diesem Patienten daher einen kausalen Zusammenhang zwischen der Hyponatriämie und den manisch-psychotischen Symptomen. Die Hyponatriämie wurde vermutlich durch exzessive Wasserzufuhr während einer Fastenperiode im Rahmen einer Wellnesskur verursacht.

## Introduction

Voltage-gated sodium and calcium channels have been implicated in the pathophysiology of bipolar disorder. Earlier reports found an association between hyponatremia and psychotic symptoms [1–4]. The following case report is intended to illustrate the importance of excluding somatic causes in the diagnosis of psychosis.

## Case presentation

We report on a 31-year old Caucasian male who was admitted to our hospital while he experienced a *manic episode with psychotic symptoms*. He presented with *rapid, incoherent speech, religious and megalomanic delusions and emotional lability*. In the days prior to admission the patient was described by relatives as behaving oddly, talking frequently about religious topics and mentioned suicidal thoughts.

The patient had no known psychiatric history except a short, most likely MDMA-induced psychotic episode some years ago. He had no relevant medical history other than a minor orthopaedic surgery and recurring Coxsackie virus infections. Family history was negative for psychiatric disorders.

At admission, blood tests were within normal ranges except for *moderate hyponatremia (127* *mmol/l, Range: 135–145* *mmol/l)*. The patient tested negative for alcohol, amphetamines, cocaine, cannabinoids, methadone, benzodiazepines, buprenorphine and other opiates. A cranial CT scan did not show any pathologies, especially no abnormalities regarding the pituitary gland. Empty sella syndrome associated with hyponatremia has previously been described presenting with manic symptoms [4]. Physical and neurological examinations did not reveal abnormal findings.

**Fig. 1:**
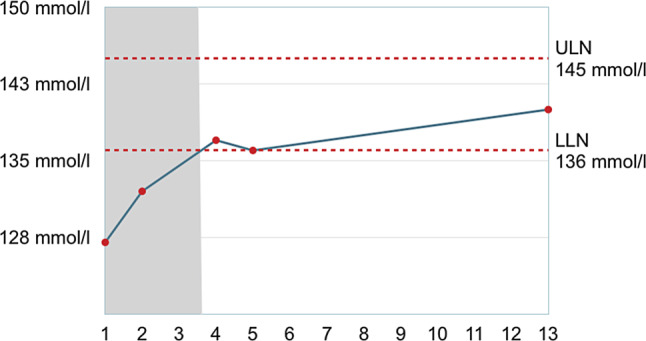
Serum sodium levels in mmol/l during stay (days 1 to 13). Dashed lines indicate upper (ULN) and lower limits of normal (LLN), according to reference range. Risperidone (*shaded area*) was discontinued on day 3, as the patient’s psychotic symptoms remitted

We started treatment with slow intravenous saline infusions and oral risperidone (3 mg/d). *Psychotic symptoms remitted within the following days, concurrently with normalization of sodium levels* (day 2: 132 mmol/l; day 5: 137 mmol/l; see Fig. 1). Risperidone was therefore discontinued on day 3. The patient stated that in the days prior to admission he had drunk large amounts of water, up to 5 or 6 l a day, as part of a spiritual wellness practice which also involved long meditation sessions.

Subsequent sodium levels during the following days continued to be within normal ranges.

The endocrinological examination as well as neurological examinations did not reveal any pathologies. After slow normalization of serum sodium levels, the patient showed no neurological signs of central pontine myelinolysis. Therefore, we decided against further MR imaging.

Since the patient’s symptoms remitted after only 3 days of treatment with risperidone while sodium levels normalized concurrently as well, *we suspected hyponatremia as the cause of psychotic symptoms and decided to discontinue antipsychotic treatment at this point*. The patient remained in the hospital for another nine days for observation.

By day 13, the patient was discharged in complete remission from psychosis and without pharmacological treatment.

## Discussion

As the *psychotic symptoms of this patient correlated with hyponatremia* we assume a causal relationship of those phenomena. In this patient, wellness-associated water intake might have led to hyponatremia inducing mania with psychotic symptoms. On the other hand, we cannot exclude prodromal symptoms as the cause for extensive water intake leading to an exacerbation of psychosis through hyponatremia. The rapid improvement of psychotic symptoms correlated clearly with the normalization of sodium levels and remained stable after discontinuation of antipsychotic treatment by day 3 until discharge at day 13.

In the literature,* dysfunction of voltage-gated ion channels* has been implicated in the pathogenesis of bipolar disorder. Most notably, genome-wide analyses have implicated two genes, CACNA1C and ANK3, of which the former encodes a subunit of a voltage-gated calcium channel and the latter encodes a protein coupling voltage-gated sodium channels to the axonal cytoskeleton [5].

Furthermore, lithium, one of the first-line treatments for acute mania, has been shown to reduce intracellular sodium and calcium levels [6, 7]. Other mood stabilizers such as valproic acid, carbamazepine, and lamotrigine, are known to act principally on voltage-gated sodium and calcium channels [8]. Given that several antiepileptic drugs which act on these channels have mood stabilizing potential as well, further research concerning the role of sodium and calcium channels in affective disorders is needed.

## Conclusion

We conclude that in this patient, there is a clear association between electrolyte disturbances and mania with psychotic symptoms, although causality cannot be definitively established. In clinical practice, we suggest to consider electrolyte disturbances as possible factors inducing new-onset psychosis.
